# Microtubules Inhibit E-Cadherin Adhesive Activity by Maintaining Phosphorylated p120-Catenin in a Colon Carcinoma Cell Model

**DOI:** 10.1371/journal.pone.0148574

**Published:** 2016-02-04

**Authors:** Stephanie L. Maiden, Yuliya I. Petrova, Barry M. Gumbiner

**Affiliations:** 1 Department of Cell Biology, University of Virginia School of Medicine, Charlottesville, Virginia, United States of America; 2 Department of Biology, Truman State University, Kirksville, Missouri, United States of America; 3 Seattle Children’s Research Institute and University of Washington School of Medicine, Seattle, Washington, United States of America; University of Colorado, Boulder, UNITED STATES

## Abstract

Tight regulation of cadherin-mediated intercellular adhesions is critical to both tissue morphogenesis during development and tissue homeostasis in adults. Cell surface expression of the cadherin-catenin complex is often directly correlated with the level of adhesion, however, examples exist where cadherin appears to be inactive and cells are completely non-adhesive. The state of p120-catenin phosphorylation has been implicated in regulating the adhesive activity of E-cadherin but the mechanism is currently unclear. We have found that destabilization of the microtubule cytoskeleton, independent of microtubule plus-end dynamics, dephosphorylates p120-catenin and activates E-cadherin adhesion in Colo 205 cells. Through chemical screening, we have also identified several kinases as potential regulators of E-cadherin adhesive activity. Analysis of several p120-catenin phosphomutants suggests that gross dephosphorylation of p120-catenin rather than that of specific amino acids may trigger E-cadherin adhesion. Uncoupling p120-catenin binding to E-cadherin at the membrane causes constitutive adhesion in Colo 205 cells, further supporting an inhibitory role of phosphorylated p120-catenin on E-cadherin activity.

## Introduction

Intercellular adhesions are critical in maintaining the integrity of developing tissues during embryogenesis as well as supporting proper tissue architecture and function in mature organisms [1,2]. The cadherin-catenin complex mediates cell-cell adhesion through calcium-dependent homophilic bonds between adjacent transmembrane cadherins [3]. This interaction is stabilized intracellularly by α-catenin, β-catenin, and p120-catenin (p120) [4,5]. β-catenin simultaneously binds α-catenin [6,7,8,9] and the cadherin cytoplasmic tail [10,11] creating a bridge to the actin cytoskeleton [12,13,14], which is critical for strong, stable adhesion [15]. p120 is a highly phosphorylated protein [16,17] that binds to the E-cadherin juxtamembrane domain [18,19,20] and is known to regulate cadherin turnover at the cell surface [21,22], providing one mechanism for controlling the level of adhesion between cells. Another way to accomplish this is by changes in cadherin gene expression [23,24], limiting the amount of cadherin available. A significant question arises, however, when cells express a complete cadherin-catenin complex but lack any adhesion to one another: how is the strength of the cadherin homophilic bond itself regulated?

There are several lines of evidence that suggest the adhesive activity of cadherin may be regulated as much as its expression. During development of *Xenopus laevis* embryos, both a dominant negative C-cadherin construct and a C-cadherin activating antibody inhibit the elongation of activin-treated animal caps [25,26], indicating that the precise adhesiveness of C-cadherin is more important during morphogenetic cell movements than its relative presence or absence. During early cell divisions of the mouse embryo, E-cadherin is expressed on the cell surface prior to the 8-cell stage, however, E-cadherin-dependent compaction of the embryo, where cell-cell adhesions first appear to engage, only occurs at the 8- to 16-cell stage [27]. A similar phenotype is seen when Colo 205 cells, a human colon carcinoma cell line, are treated with either the kinase inhibitor staurosporine, low levels of trypsin [28], or specific monoclonal antibodies to the E-cadherin ectodomain [29]. Under these conditions, the normally rounded and dispersed cells clump together and compact, causing individual cells to no longer be discernable. These various examples suggest that an intracellular signaling cascade may be able to alter the extracellular adhesive activity of E-cadherin during specific cellular events.

p120 has emerged as an important component of this inside-out signaling pathway regulating cadherin adhesive function. In the conditions described above that trigger adhesion in Colo 205 cells, p120 is known to be dephosphorylated [28,29], and when a phosphorylation-deficient p120 mutant is expressed, Colo 205 cells become constitutively adhesive [29]. Adhesion activation in Colo 205 cells also causes the unmasking of an epitope near the p120 binding site of E-cadherin, which can be observed with an antibody to the E-cadherin cytoplasmic tail [29]. Couple this fact with the isolation of monoclonal E-cadherin antibodies that either distinguish active and non-active E-cadherin, or that can trigger E-cadherin adhesion themselves [29], and conformational control of E-cadherin seems highly likely. A similar mechanism has been described for integrin regulation in extracellular matrix adhesion [30,31,32] but the molecular components that may regulate E-cadherin in such a way remain to be determined. The current hypothesis is that the phosphorylation state of p120 may act as a molecular switch to control the adhesive activity of cadherin.

p120 is a member of the armadillo-repeat family of proteins [33] and also has N-terminal coiled-coil and regulatory domains [34]. Within the regulatory domain lies a phosphorylation domain that harbors eleven tyrosine, serine, and threonine phosphorylation sites [16,17]. There is evidence that protein kinase C modulates phosphorylation at these sites [16], however, protein kinase C activation in Colo 205 cells had no affect on the adhesive state of E-cadherin [28]. While several Src-family kinases have been associated with p120 [35,36,37], very few serine/threonine kinases have been identified despite the predominance of serine/threonine phosphorylation [16]. p120 binds cadherin through its armadillo repeats [38] and most likely stabilizes cell surface expression of cadherin by inhibiting the early stages of endocytosis [21,22]. Because cell surface expression of E-cadherin does not change upon adhesion activation in Colo 205 cells [28,29], changes in endocytosis are unlikely to be the cause. The microtubule cytoskeleton has also been shown to interact with p120 through both direct and indirect mechanisms [39,40] but the function of such an interaction has not been well studied. Microtubules have an inherent polarity, with microtubule elongation occurring more rapidly at the plus-end rather than the minus-end [41], and there have been reports that either end may interact with cadherin-catenin adhesions through a variety of microtubule-associated proteins [39,42,43,44]. Microtubules could provide structural support to cadherins during activation or could be responsible for trafficking the molecular mediators that regulate p120 phosphorylation.

The aim of this study was to identify factors that regulate p120 phosphorylation in relation to changes in E-cadherin adhesive activity. Through this work we have found that the microtubule cytoskeleton inhibits E-cadherin adhesive activity by maintaining phosphorylated p120 in Colo 205 cells. Using a chemical approach, we have also identified several serine/threonine kinases as potential regulators of E-cadherin adhesive activity. Through site-directed mutagenesis, we have also found that the overall amount of dephosphorylation of p120 rather than that of specific amino acids may trigger E-cadherin adhesion. Uncoupling p120 binding to E-cadherin at the membrane causes constitutive adhesion in Colo 205 cells, further supporting an inhibitory role of phosphorylated p120 on E-cadherin activity.

## Results

### Destabilizing Microtubules Stimulates Cadherin Adhesion through Dephosphorylation of p120

**Fig 1:**
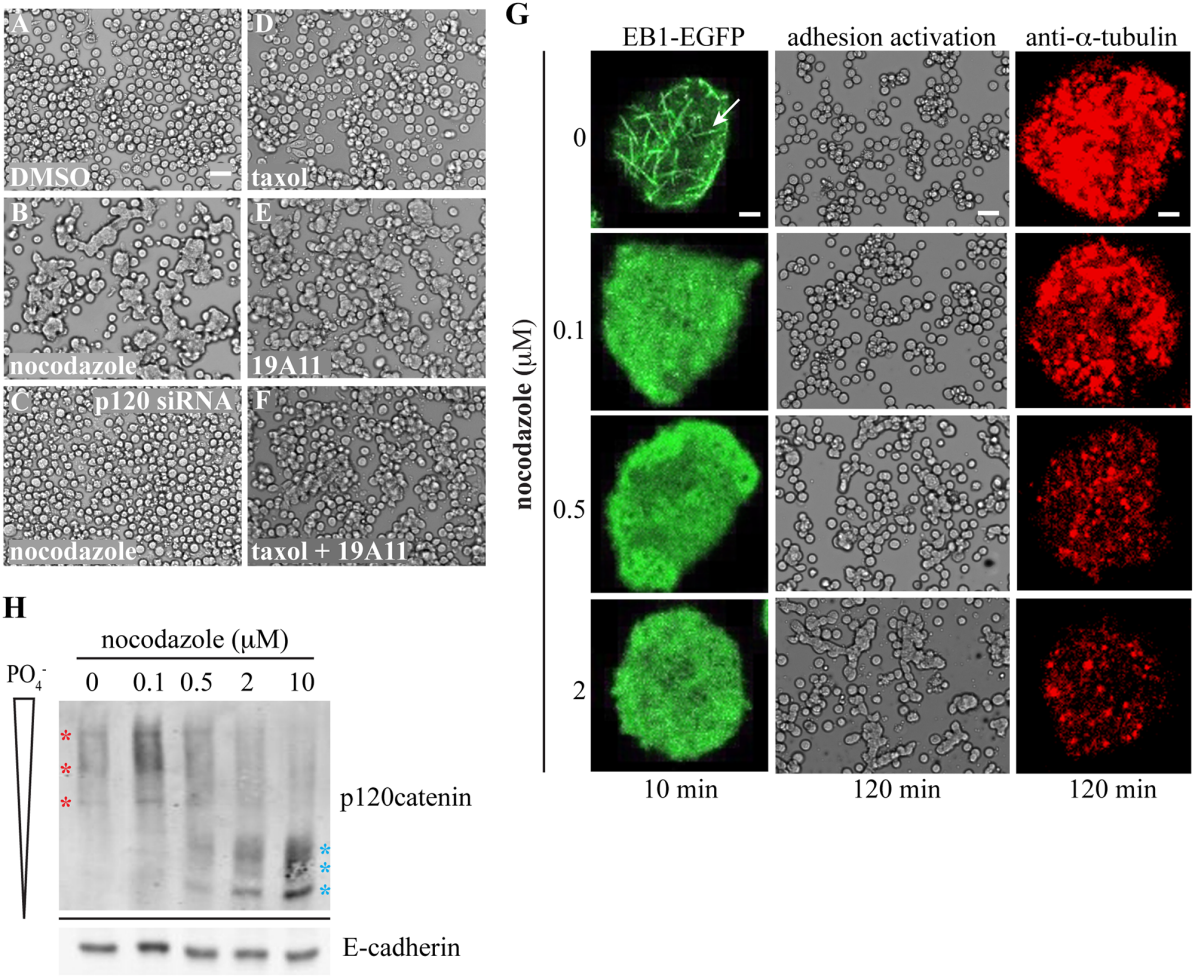
Microtubule disruption dephosphorylates p120 and stimulates cadherin-based adhesion in Colo 205 cells. (A-F) Brightfield microscopy of non-adhesive and adhesion-activated Colo 205 cells. Scale bar = 50 μm. (A-C) Parental or p120 knockdown cells were treated with either 10 μM nocodazole or an equal volume of DMSO for 1.5 hours. (D-F) Parental cells were pre-treated with 10 μM taxol for 2 hours then treated with 2 μg/mL 19A11 activating E-cadherin mAb for 4 hours. (G) Colo 205 cells expressing EB1-EGFP were treated with various concentrations of nocodazole and imaged at 10 minutes via fluorescent confocal microscopy. Scale bar = 5 μm. The same cells were then imaged by brightfield microscopy (scale bar = 50 μM) after 2 hours of treatment to examine the extent of adhesion activation, then fixed in methanol and immunostained for α-tubulin to examine the extent of microtubule disruption (scale bar = 5 μm). (H) Cells were treated for 1.5 hours with various concentrations of nocodazole then lysed in 1% triton x-100. Samples were resolved on 6% SDS-PAGE with 20 μM Phos-tag reagent then visualized via Western blotting. Red stars = higher molecular weight in non-activated samples, cyan stars = lower molecular weight in activated samples.

Microtubule polymerization occurs more quickly at its plus-end, which exhibits cycles of shrinkage (catastrophe) and growth (rescue) known as dynamic instability. The slow-growing microtubule minus-ends tend to be anchored at microtubule organizing centers like the centrosome [41]. Both ends of the microtubule have been found associated with cadherin-catenin adhesions through different microtubule-associated proteins [39,40,42,43,44] but the functional significance of such an interaction in either case is not well understood. Therefore, we wanted to determine if perturbation of the microtubule network would have an effect on the activation of E-cadherin adhesion. When compared to DMSO-treated control cells, which remain rounded and dispersed (Fig 1A), Colo 205 cells treated with the microtubule destabilizing drug nocodazole exhibit both cell aggregation and compaction (Fig 1B). This effect is blocked when the endogenous p120 is depleted by siRNA (Fig 1C). This 95% reduction in p120 simultaneously depletes E-cadherin almost 60% [29] because p120 binding is important in blocking key sequences for the endocytosis of E-cadherin [21,22]. This decrease in E-cadherin does not allow us to test the requirement for p120 in activation but at least shows the specificity of the nocodazole effect on cadherin-catenin adhesion rather than another adhesion system. Surprisingly, cells treated with taxol, a microtubule stabilizing drug, do not phenocopy the effects of nocodazole (Fig 1D), nor is it able to block the morphology changes caused by addition of an activating E-cadherin monoclonal antibody, 19A11 (Fig 1E and 1F) (see reference [29] for antibody description). Since both nocodazole and taxol should disrupt dynamic instability, this data supports a model whereby microtubules inhibit cadherin adhesive activity independent of microtubule plus-end dynamics.

To test whether or not microtubule plus-end dynamics play a role in inhibiting cadherin activation, we expressed a microtubule plus-end reporter, EB1-EGFP, in Colo 205 cells and analyzed dynamic instability upon treatment over a range of nocodazole concentrations (Fig 1G). With DMSO only (0 μM nocodazole), live imaging of the EB1-EGFP reporter revealed growing microtubule plus-ends by visualization of characteristic comets (arrow) [45]. After two hours, these control cells remained non-adhesive and further immunostaining found the vast microtubule network to be intact. When treated with a low dose of nocodazole (0.1 μM), which is thought to only perturb plus-ends, EB1-EGFP comets disappeared completely within 10 minutes. However, after two hours, the cells remained non-adhesive and microtubules were mostly maintained. At 0.5 μM nocodazole, small compacted cell aggregates can be seen and the microtubule network is more visibly disrupted. At 2 μM nocodazole, cell aggregation and compaction is obvious and the overall microtubule network is perturbed. From this data, we conclude that disruption of microtubule plus-ends alone is not sufficient to drive activation of cadherin adhesion in Colo 205 cells.

Previous work has shown the importance of p120 dephosphorylation in driving cadherin adhesion activation [28,29]. Given the effects of nocodazole in Colo 205 cells (Fig 1B), we wanted to analyze the state of p120 phosphorylation upon treatment with nocodazole (Fig 1H). Initial results with SDS-PAGE and Western blotting did show a small decrease in the molecular weight of p120 upon adhesion activation with nocodazole, which is indicative of dephosphorylation (data not shown). To look at this more carefully, however, we decided to utilize Phos-tag^™^ acrylamide during our SDS-PAGE, a novel phosphate-binding molecule that will further resolve proteins depending on the degree of phosphorylation [46]. At non-activating doses of nocodazole (0, 0.1 μM; also see Fig 1G), we see several higher molecular weight bands for p120 (red stars) than we do at doses where adhesion activation is readily apparent (2, 10 μM; cyan stars; also see Fig 1G). At 0.5 μM nocodazole, where adhesion activation is not as robust (Fig 1G), we can see both high molecular weight bands (red stars) as well as the lower molecular weight bands (cyan stars, Fig 1H). We also found no overt changes in E-cadherin expression between non-activating and activating doses of nocodazole (Fig 1H, lower panel). From this analysis, we conclude that p120 is dephosphorylated at concentrations of nocodazole sufficient to trigger adhesion activation in Colo 205 cells.

### Chemical Perturbation of Certain Serine/Threonine Kinases Can Activate Cadherin Adhesion

**Fig 2:**
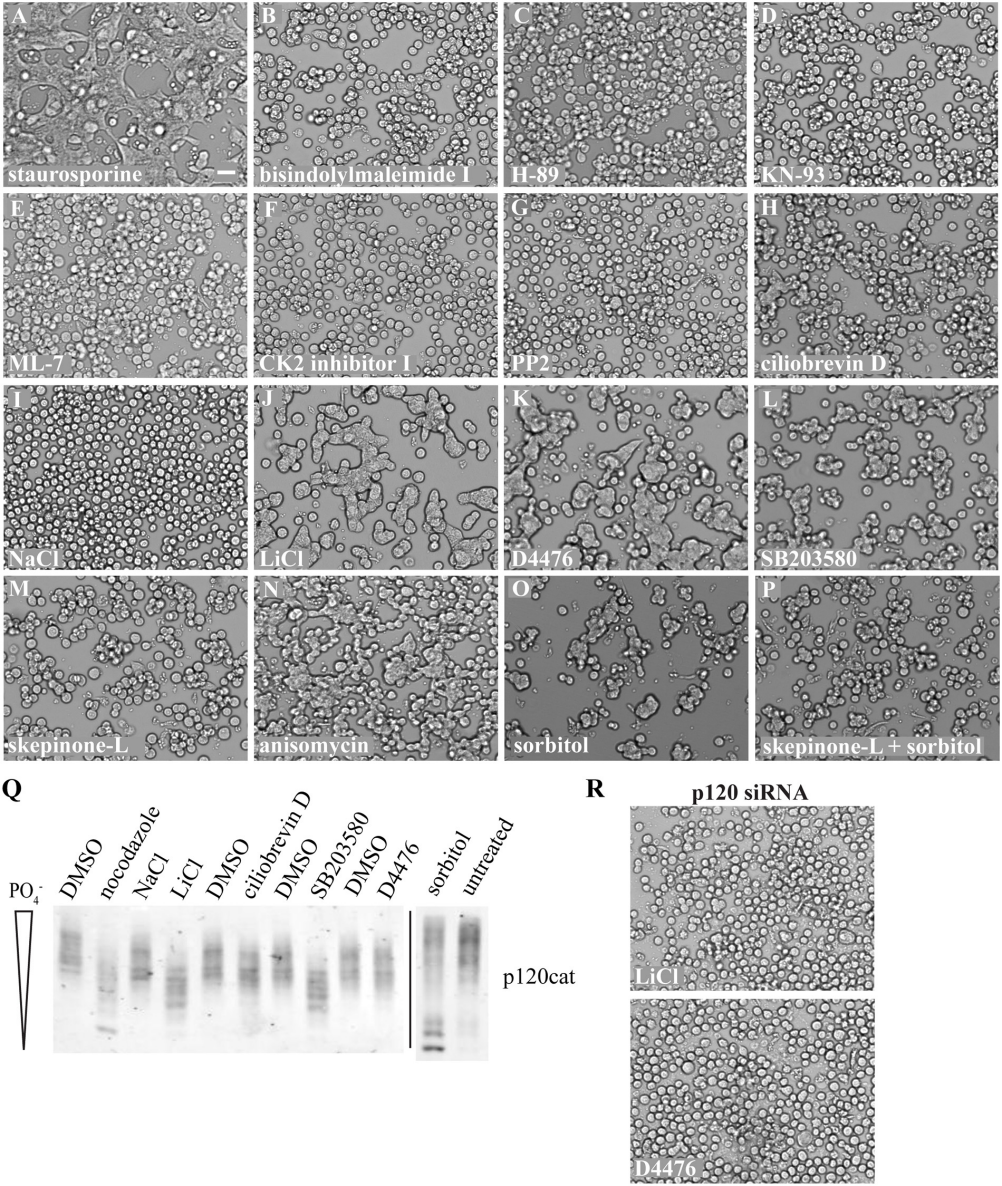
Chemical perturbation of certain serine/threonine kinases activates cadherin adhesion in Colo 205 cells. (A-P) Cells were treated with various drugs known to inhibit ser/thr kinases at a range of concentrations for 1.5–6 hours (S1 Table). If cells remained non-adhesive, the brightfield image shown corresponds to the highest dose tested. If cells became adhesive, the lowest dose tested with an effect is shown. Scale bar = 50 μm. (Q) Cells that became adhesive were lysed in 1% triton x-100 and resolved by 6% SDS-PAGE with 20 μM Phos-tag reagent. The extent of p120 dephosphorylation was then visualized via Western blotting. (R) Brightfield images of cells treated with either 55 mM LiCl or 50 μM D4476 after knockdown of p120.

To elucidate other molecular components that may be regulating E-cadherin adhesive activity, we screened a variety of chemicals to perturb certain pathways possibly related to cadherin-catenin adhesion (Fig 2 and S1 Table). Staurosporine is a known activator of adhesion in Colo 205 cells (Fig 2A) [28,29] but it inhibits a broad range of serine/threonine kinases, including protein kinase C, protein kinase A, calcium/calmodulin-dependent kinase II, and myosin light chain kinase. Using more specific inhibitors to these kinases, we sought to identify the individual kinases responsible for regulating adhesion activation. Unfortunately, neither bisindolylmaleimide I (protein kinase C), H-89 (protein kinase A), KN-93 (calcium/calmodulin-dependent kinase II), nor ML-7 (myosin light chain kinase) treatment triggered adhesion activation in Colo 205 cells (Fig 2B–2E). We recently showed that lithium chloride, a well-known inhibitor of glycogen synthase kinase 3 (GSK3), activates adhesion in Colo 205 cells when compared to sodium chloride alone (Fig 2I and 2J) [47]. Because of a link to GSK3 through the Wnt signaling pathway [48], we also tested an inhibitor to casein kinase 2 (Fig 2F) but observed no effect on Colo 205 morphology. Even though p120 is predominantly phosphorylated at serine/threonine residues under normal conditions [16], it was originally identified as a Src substrate and can be tyrosine phosphorylated [4,17,33]. To determine if Src-family kinases may be regulating E-cadherin activity through p120, we also treated cells with the PP2 inhibitor (Fig 2G) but again observed no effect.

Due to our result that microtubules inhibit E-cadherin activity independent of plus-end dynamics (Fig 1), we also treated cells with ciliobrevin D (Fig 2H), which inhibits the microtubule minus-end directed motor protein cytoplasmic dynein [49]. After 6 hours, these cells exhibited some minor clumping but did not phenocopy nocodazole-treated cells (Fig 1B) or activating antibody-treated cells (Fig 1E). An inhibitor to casein kinase 1 (CK1), D4476, was also tested since several reports have shown a dual phosphorylation mechanism with GSK3 [50]. Like lithium chloride treatment (Fig 2J), treatment of Colo 205 cells with the D4476 inhibitor resulted in robust activation of adhesion (Fig 2K). We also found evidence in the literature that the activity of p38 MAPK may be regulated by an interaction with microtubules [51] and that p38 MAPK may inhibit GSK3 [52]. We therefore treated cells with the well-known p38 MAPK inhibitor SB203580 and discovered strong cell clumping and compaction (Fig 2L). After further research, however, we found a report showing that SB203580 also inhibits CK1 [53], so we utilized a more specific inhibitor, skepinone-L [54]. Treatment with this inhibitor did not activate adhesion (Fig 2M). p38 MAPK is generally involved in signaling cascades that respond to cell stress and becomes activated by phosphorylation at two adjacent residues [55]. We decided to also treat cells with compounds known to activate p38 MAPK and found that both anisomycin and sorbitol [51,56] caused cells to clump and compact (Fig 2N and 2O). We also found that the effect of sorbitol was abrogated by pre-treatment of cells with the p38 MAPK inhibitor skepinone-L (Fig 2P).

Since dephosphorylation of p120 appears to be a critical event in activating cadherin adhesion, we decided to analyze the extent of p120 phosphorylation after these novel activating treatments by utilizing Phos-tag SDS-PAGE [46]. In Shashikanth *et*. *al*., we showed through normal SDS-PAGE analysis that treatment of Colo 205 cells with lithium chloride resulted in a shift to a lower molecular weight for p120, which is indicative of dephosphorylation [47]. This dephosphorylation can be more clearly seen with the Phos-tag compound (Fig 2Q), which reveals several shifted bands compared to sodium chloride-treated control cells. Lithium chloride treatment does not appear as robust as nocodazole in dephosphorylating p120 but appears similar to SB203580 (Fig 2Q). Ciliobrevin D was also analyzed due to the minor clumping seen after treatment, but this compound did not dephosphorylate p120 appreciably. Sorbitol treatment strongly dephosphorylates p120 most similar to nocodazole. Therefore, activated p38 MAPK must affect p120 indirectly and act upstream of its dephosphorylation.

Surprisingly, D4476 does not appear to alter p120 phosphorylation even though treatment strongly activates cell adhesion. Since the CK1 inhibitor D4476 is the only adhesion activator that does not appear to dephosphorylate p120, we sought confirmation that the morphology effects seen were at least due to cadherin-based adhesion. Like the effect on nocodazole treatment (Fig 1B and 1C), p120 and E-cadherin knockdown with a p120 siRNA completely blocked the effects of lithium chloride and D4476 treatment on Colo 205 cells (Fig 2R). Therefore, CK1 may act downstream of p120 in inside-out signaling or in a parallel pathway that can regulate E-cadherin activity independent of p120 dephosphorylation. In conclusion, the serine/threonine kinases GSK3, CK1 and p38 MAPK may represent a subset of signaling molecules that participate in the regulation of E-cadherin adhesiveness.

### Overall p120 Phosphorylation Is Important in Regulating Cadherin Activity

**Fig 3:**
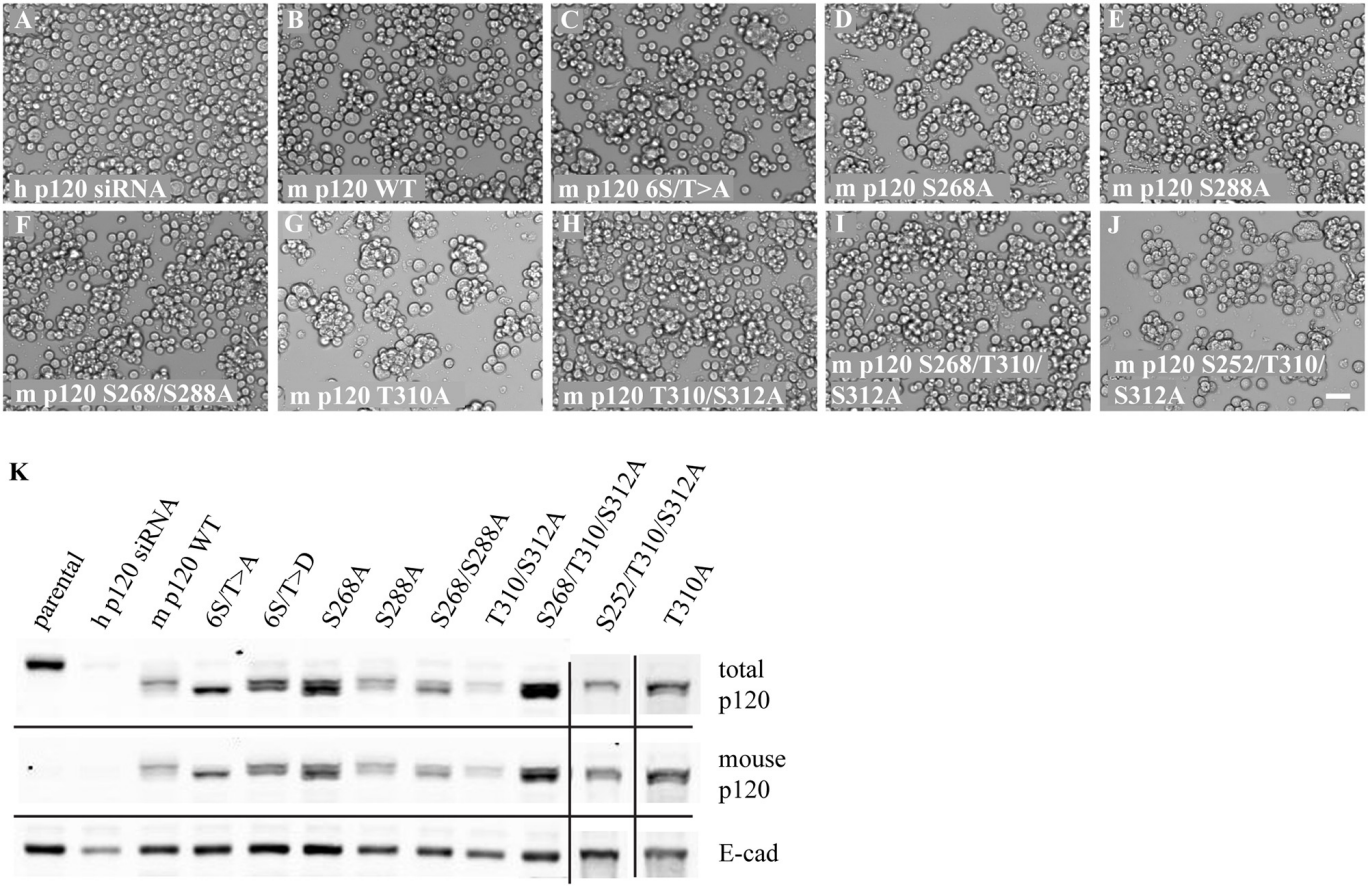
The overall state of p120 phosphorylation regulates cadherin adhesive activity. (A-J) Brightfield images of Colo 205 cells expressing mouse p120 non-phosphorylatable mutants with knockdown of endogenous human p120 by siRNA. Scale bar = 50 μm. (K) Cells in A-J were lysed in 1% triton x-100, resolved on 6% SDS-PAGE, and visualized via Western blotting.

In Petrova *et*. *al*., we showed that expression of a phosphorylation-deficient mutant of p120 causes constitutive adhesion in Colo 205 cells. Six serine/threonine residues were mutated to alanine to create this mutant but several individual mutations also exhibited effects on cell clumping, albeit to a lesser extent [29]. Based on these results, we sought to determine whether or not specific combinations of dephosphorylated residues can regulate cadherin activity. When the endogenous human p120 is depleted, Colo 205 cells remain rounded and dispersed (Fig 3A) similar to untreated cells. If a wildtype mouse p120 construct is expressed in this background (Fig 3B), the cells exhibit a minor amount of clumping compared to the control (Fig 3A), but no significant compaction. However, expression of the phosphorylation-deficient mutant 6S/T>A results in a high degree of cell clumping as well as compaction (Fig 3C). As we previously published in Petrova, *et al*., a p120 S268A mutant also exhibits a high degree of cell clumping but not compaction (Fig 3D), and a S288A mutant exhibits more cell clumping than the wildtype mouse p120 but less than that of S268A (Fig 3E) [29]. Expression of these two mutations in tandem, however, does not appear to increase the clumping or compaction of these cells when compared to either mutation alone (Fig 3F). While Western blot analysis utilizing a p120 phospho-specific antibody to residue T310 showed dephosphorylation at this site upon adhesion activation, a non-phosphorylatable mutant had not been analyzed [29]. Expression of mouse p120 T310A did result in clumping of Colo 205 cells (Fig 3G) similar to the S268A mutant (Fig 3D). This mutation was expressed in tandem with S312A, a mutant previously shown to have minor affects on adhesion activation, but there was no noticeable increase in clumping or compaction observed (Fig 3H). Expression of a triple mutant consisting of S268A, T310A, and S312A also did not cause adhesion activation greater than the individual mutations alone (Fig 3I). An alternative triple mutant consisting of S252A, T310A, and S312A was also created based on evidence that GSK3 is able to phosphorylate those residues *in vitro*, but again, no increase in clumping or compaction relative to the individual mutations was observed (Fig 3J). These results are summarized in Table 1. While there is some variability in the expression of each mutant (Fig 3K), nocodazole treatment confirmed the ability of the cells to be strongly activated (data not shown), ruling out insufficient E-cadherin or p120 as a cause for less robust effects. In summary, we find that the overall phosphorylation status of p120, rather than that of specific amino acids, is most important in determining the activity state of E-cadherin.

**Table 1:** Level of adhesion in non-phosphorylatable p120 mutants.

cells	adhesion	cells	adhesion
h p120 siRNA	-	S268/S288A	++
m p120 WT	+	T310A	+++
6S/T>A	++++	T310/S312A	++
S268A	+++	S268/T310/S312A	++
S288A	++	S252/T310/S312A	++

Qualitative assessment of adhesion in p120 non-phosphorylatable mutants by comparing the amount of clumping and the level of compaction between cells. (-) indicates non-adhesive, dispersed cells; (+) indicates <10% of cells clump but do not compact; (++) indicates ≤50% of cells clump but do not compact; (+++) indicates ≥50% of cells clump but <10% compact; (++++) indicates ≥50% of cells clump and the majority of those are compacted.

In order to understand additional mechanisms that may regulate cadherin adhesive activity, we attempted to utilize phosphomimetic p120 mutants that would block cadherin adhesion activation. Previous work in the lab had described one such p120 mutant, 4S/T>E, which results in glutamic acid substituting for serine/threonine residues [29], but expression of this particular mutant was problematic. In attempts to rectify this, it was discovered that the expression plasmid had an additional mutation in the p120 stop codon, which presumably allowed for additional amino acids to be translated from the vector sequence. When this was corrected, however, the 4S/T>E phosphomimetic mutant no longer blocked adhesion activation in Colo 205 cells (Table 2; S1 Fig). The level of clumping and compaction in p120 4S/T>E expressing cells was similar to wildtype in both untreated cells and those treated with 19A11 activating antibody, nocodazole, or lithium chloride. Further mutation of residues S252 and T910 to create 6S/T>E, which would match the amino acids mutated in the constitutively adhesive 6S/T>A mutant, also did not block adhesion activation when expressed in Colo 205 cells (data not shown). While glutamic acid (E) is often used to mimic phosphorylated residues, so is aspartic acid (D). Unfortunately, expression of p120 6S/T>D (Fig 3K) was also not able to block adhesion activation by the three treatments tested (Table 2 and S1 Fig).

**Table 2:** Activation of adhesion in p120 phosphomimetic mutants.

cells	19A11 activating Fab	nocodazole	LiCl
h p120 siRNA	**X**	**X**	**X**
m p120 WT	✔	✔	✔
m p120 6S/T>A	✔	✔	✔
m p120 6S/T>D	✔	✔	✔
m p120 4S/T>E	✔	✔	✔

Assessment of adhesion activation by comparing the amount of clumping and the level of compaction between cells treated with 2 μg/mL 19A11 activating E-cadherin Fab, 10 μM nocodazole, or 55 mM LiCl.

**Fig 4:**
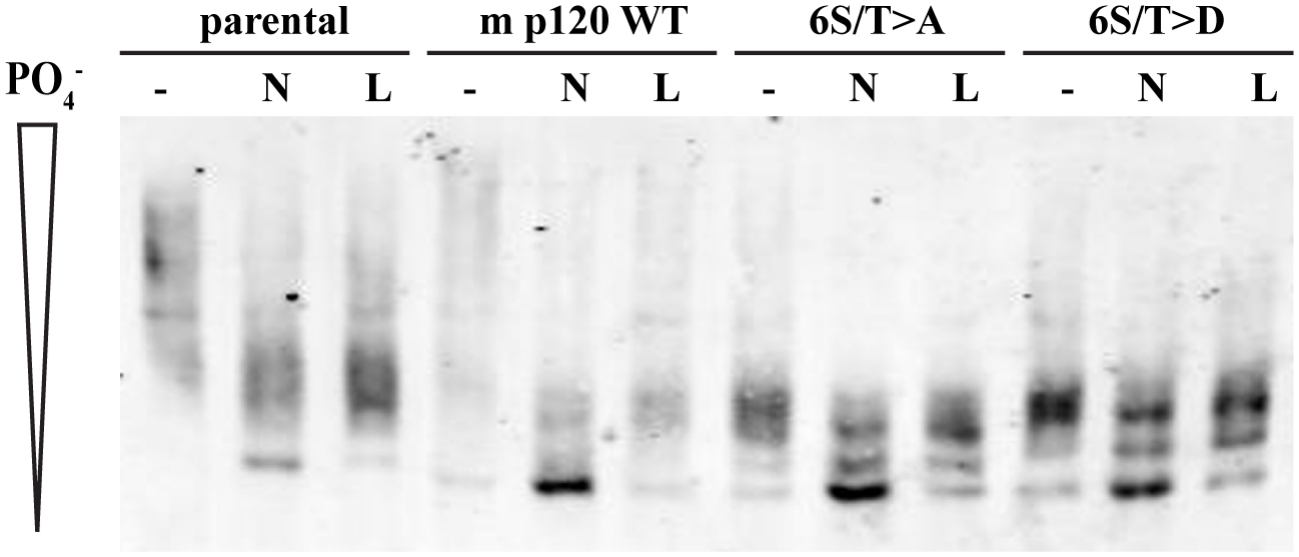
Additional phosphorylated residues of p120 may contribute to the regulation of cadherin-based adhesion. Colo 205 cells expressing various mouse p120 phosphomimetic mutants with endogenous human p120 knockdown by siRNA before treatment. Untreated (-), 10 μM nocodazole (N), and 55 mM LiCl (L) treated cells were lysed in 1% triton x-100 and resolved by 6% SDS-PAGE with 20 μM Phos-tag reagent. The extent of p120 dephosphorylation was then visualized via Western blotting.

While the phosphorylation-deficient p120 mutant 6S/T>A appears constitutively adhesive when expressed in Colo 205 cells, we observed further compaction of cell clumps upon treatment with either nocodazole or lithium chloride (S1 Fig). We hypothesized that these treatments may dephosphorylate additional amino acids not mutated in the 6S/T>A construct. To test this hypothesis, we again utilized Phos-tag SDS-PAGE analysis (Fig 4). As also shown in earlier figures, treatment of parental Colo 205 cells with either nocodazole (N) or lithium chloride (L) causes the p120 bands to shift to lower molecular weights compared to untreated controls (-), and the same trend can be seen in cells expressing the wildtype mouse p120. In lysates from untreated cells expressing mouse p120 6S/T>A, we see that the p120 bands are already at lower molecular weights than both untreated wildtype mouse p120 and parental cells, owing to the constitutive adhesion observed with this construct. When 6S/T>A is treated with nocodazole, the p120 bands are shifted even further. The same trend is seen with lithium chloride treatment but to a lesser extent. We also analyzed the 6S/T>D mutant, which should still block phosphorylation at the same amino acids as 6S/T>A, and observed the same results. In conclusion, there appear to be additional unknown phosphorylated amino acids of p120 that may contribute to robust cadherin adhesion activation in Colo 205 cells.

### Uncoupling E-Cadherin and p120 Causes Constitutive Adhesion in Colo 205 Cells

**Fig 5:**
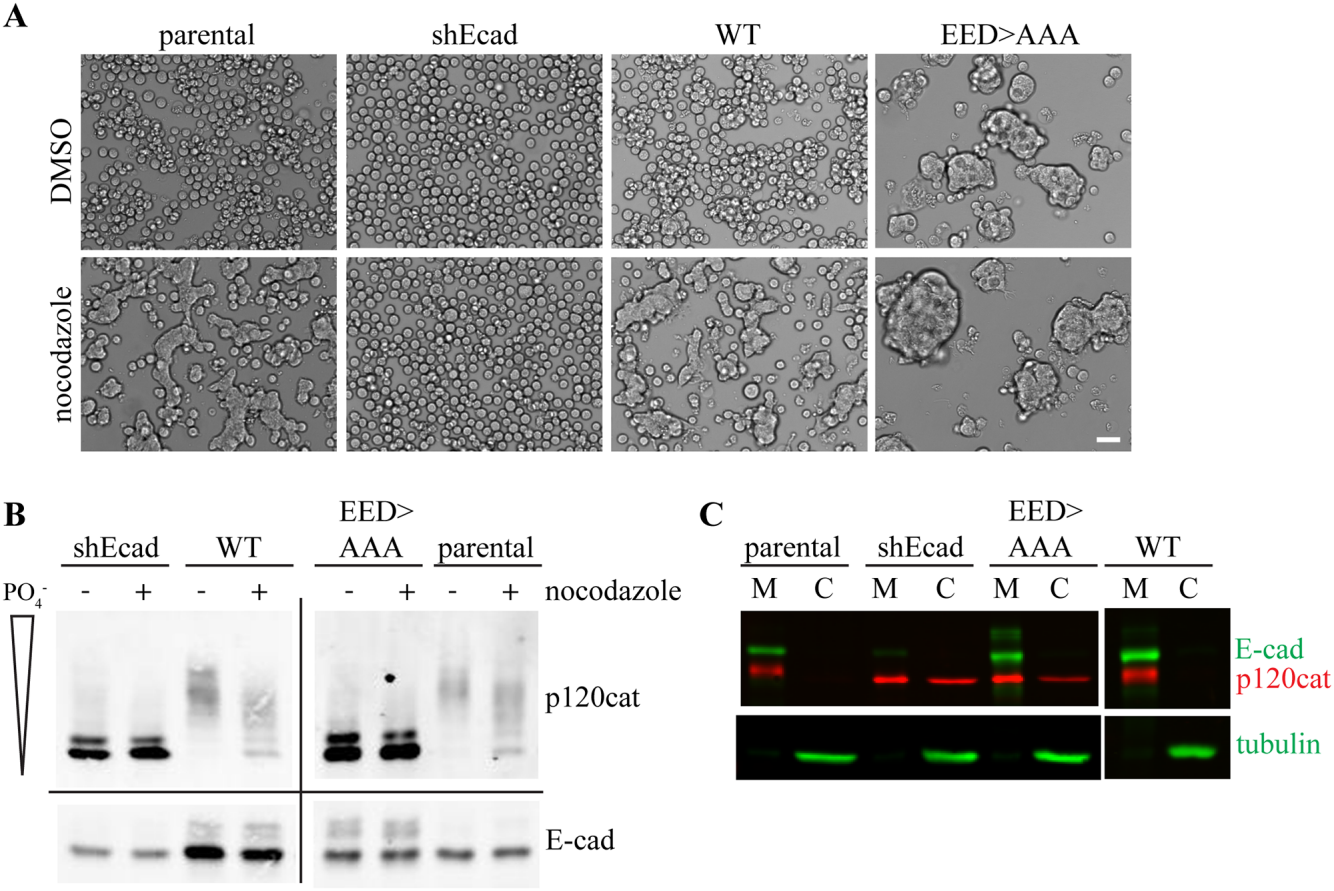
A p120-binding deficient E-cadherin mutant exhibits constitutive adhesion in Colo 205 cells. Colo 205 cells expressing human E-cadherin constructs behind stable knockdown of endogenous human E-cadherin by shRNA specific to an untranslated region. (A) Brightfield images of cells treated with either 10 μM nocodazole or an equal volume of DMSO. Scale bar = 50 μm. (B) Cells were lysed in 1% triton x-100 and resolved by 6% SDS-PAGE with 20 μM Phos-tag reagent. The extent of p120 dephosphorylation and E-cadherin expression was then visualized via Western blotting. (C) Subfractionation of cells in hypotonic buffer resolved by 7.5% SDS-PAGE and visualized by Western blotting. M = membrane, C = cytosol.

As discussed earlier, p120 knockdown also depletes E-cadherin, which has made it difficult to address whether or not binding of dephosphorylated p120 to E-cadherin is required to initiate adhesion activation. To determine this, we expressed a p120-binding deficient E-cadherin mutant, EED>AAA [18], in Colo 205 cells with stable shRNA knockdown of endogenous E-cadherin (Fig 5A). Because this p120-binding deficient E-cadherin may also be removed from the cell surface more quickly due to increased endocytosis [21,22], we used flow cytometry to carefully measure the cell surface expression of E-cadherin before all experimentation (data not shown). While this mutant showed robust cell surface expression for at least one week post-selection, expression was eventually lost with further cell passages and stable lines could not be maintained. All experiments discussed herein were conducted with cells that had EED>AAA E-cadherin cell surface expression comparable to parental E-cadherin levels.

Compared to parental cells, the E-cadherin knockdown cells (shEcad) do not respond to nocodazole treatment (Fig 5A). When wildtype E-cadherin is reconstituted, the cells exhibit strong adhesion activation in the presence of nocodazole. With expression of E-cadherin EED>AAA, however, the DMSO-treated control cells exhibit robust clumping and compaction on their own, and treatment with nocodazole does not appear to cause any further morphology changes. This result is similar to Colo 205 exogenous expression of an N-cadherin mutant missing the juxtamembrane domain (amino acids 761–842) [28]. Using Phos-tag SDS-PAGE (Fig 5B), we see that the p120 bands in shEcad Colo 205 cells migrate at the two lowest molecular weight bands seen in both the DMSO and nocodazole-treated cells. This is in stark contrast to wildtype E-cadherin, which shows higher molecular weight p120 bands shifting upon nocodazole treatment. Similar to the E-cadherin knockdown cells, expression of the E-cadherin EED>AAA mutant results in p120 bands migrating at the two lowest molecular weights observed regardless of treatment. Since it is well-known that p120 must be associated with the membrane to be phosphorylated, this data is consistent with E-cadherin EED>AAA being unable to bind p120. To confirm, we performed a cell fractionation experiment to analyze whether or not p120 was membrane-bound (Fig 5C) [18]. In parental Colo 205 cells, both E-cadherin and p120 appear in the membrane fraction (M) rather than in the cytosol (C). In E-cadherin knockdown cells, however, a large portion of p120 can be found in the cytosolic fraction. With expression of E-cadherin EED>AAA, the distribution of p120 between the membrane and cytosolic fractions is similar to the E-cadherin deficient cells, confirming p120 appears unbound relative to E-cadherin. While it is unclear what other protein may be recruiting a portion of p120 to the membrane in the E-cadherin knockdown cells, the p120 present does not appear to be phosphorylated (Fig 5B). We conclude that when not bound to p120, E-cadherin appears to be constitutively activated, resulting in robust adhesion in Colo 205 cells.

## Discussion

While textbook depictions of intercellular adhesions may leave the impression that such protein complexes are static, mounting evidence suggests they are in fact extremely dynamic. During development, cadherin-mediated adhesions must simultaneously maintain tissue integrity while still allowing for the cell rearrangements that pattern the embryo. Adhesive connections must therefore be highly adaptable and adjust in strength and number based on the needs of the developing tissue [1,2]. In an epithelium, the cadherin-catenin complex can usually be found concentrated apically in the lateral membranes between cells connecting to a belt of actin and forming the adherens junction [57]. Previous studies concerning cadherin-based adhesion tend to focus on adherens junction continuity or organization and not directly on adhesion, partly because the two are so hard to distinguish. There are key properties of Colo 205 cells that make them extremely useful for studying the initiation of adhesion over the stability of the adherens junction. While the cells are epithelial in origin and express cadherin-adhesion components [28], the proteins are not polarized and remain dispersed throughout the apical, basal, and lateral membranes, even after adhesion activation (unpublished observations). By using this cell line, we are able to provide further evidence of an inside-out signaling pathway that regulates the adhesive activity of E-cadherin. We focused on how this may occur through p120 since p120 dephosphorylation is concomitant with adhesion activation in Colo 205 cells [28, 29]. While our results shown here support the hypothesis that the overall phosphorylation state of p120 is most important in determining the activity state of cadherin, we can not rule out the possibility that the sequential order in which individual sites are phosphorylated or dephosphorylated is also important. It is also well known that p120 regulates E-cadherin turnover [21,22], but since changes in E-cadherin expression do not occur between different activation states in Colo 205 cells [28,29] (Fig 1H), it can not be the cause of the change in adhesive strength. p120 is also known to affect the activity of Rho-GTPases, which have diverse functions in both cell-cell and cell-matrix adhesions owing to their ability to alter the actin cytoskeleton [58]. Unfortunately, expression of a dominant negative or a constitutively active RhoA in Colo 205 cells did not initiate or block adhesion activation in our hands. A previously described p120 mutant unable to bind RhoA [59] was also expressed in Colo 205 cells but we did not find that this construct affected adhesion activation (data not shown).

In this paper, we show that microtubule destabilization activates adhesion in Colo 205 cells and dephosphorylates p120. Direct binding of microtubules to p120 has been shown to occur through the armadillo repeats and is therefore mutually exclusive to cadherin binding [40]. Indirect association to p120 can occur through either kinesin, a microtubule plus-end directed motor [40], IQGAP bound to E-cadherin [43], or PLEKHA7 bound to Nezha, a minus-end binding protein [39]. Both kinesin and PLEKHA7 bind the N-terminus of p120-catenin [39,40] and are therefore attractive candidates as proteins involved in inside-out regulation of cadherin. Unfortunately, siRNA knockdown of a number of microtubule-associated proteins, including IQGAP1, CLIP-170, kinesin, KIF5B, PLEKHA7, and even EB1, did not cause adhesion activation in Colo 205 cells, nor block activation by other methods (data not shown). Since we found that perturbation of plus-end dynamics alone is not sufficient to initiate adhesion activation (Fig 1), the lack of results from knockdown of plus-end proteins like IQGAP1 and CLIP-170 is not surprising, but other than Nezha, there are not many well described minus-end binding proteins. Trafficking along microtubules to spatially and temporally regulate molecular mediators involved in cadherin activation is still a viable hypothesis, but knockdown of the kinesins mentioned above, and even inhibition of cytoplasmic dynein (Fig 2H), had no affect on adhesion activation. Our identification of casein kinase 1 and p38 MAPK as potential regulators of cadherin activity are compelling due to various reports linking these serine/threonine kinases to microtubule trafficking. Casein kinase 1 was found to activate dynein-dependent minus-end directed transport of pigment granules in *Xenopus laevis* [60]. If CK1 inhibition were to halt similar transport mechanisms in Colo 205 cells, that would explain why such inhibition phenocopies destabilization of microtubules by nocodazole. p38 MAPK activation was previously shown to require both dynein and an intact microtubule cytoskeleton [51], which would appear to contradict our result that activation of p38 MAPK also phenocopies nocodazole treatment. However, this difference could be a matter of context and our results support further exploration into the role of p38 MAPK in the regulation of cadherin activation.

In stably adhesive cells, it has been reported that recruitment of microtubules to the cadherin-catenin complex supports strong adhesion by maintaining cadherin expression and distribution in the membrane [44], but our results with nocodazole-induced adhesion in Colo 205 cells implies the mechanism for cadherin activation must be distinct. It is also interesting to note that nocodazole treatment of constitutively adhesive Colo 205 cells, like those expressing the phosphorylation-deficient p120 mutant 6S/T>A (Fig 4A and S1 Fig) or E-cadherin EED>AAA (Fig 5A), did not disrupt those adhesions; however, Colo 205 cells do not exhibit *bona fide* adherens junctions like others of epithelial origin. Nevertheless, there is accumulating evidence that this mechanism of cadherin regulation is not specific to Colo 205 cells. We previously published that two growth factor induced processes, tubulogenesis in MDCK cells and wound healing in A431 cells, which require the downregulation of E-cadherin adhesion, could be inhibited with an activating E-cadherin monoclonal antibody [29]. We also demonstrated that expression of the constitutively active mouse p120 6S/T>A mutant in A431 cells significantly increased adhesion over already adhesive control cells [29]. Other investigators have found that the HT-29 epithelial cell line exhibits p120 band shifts upon epithelial sheet formation similar to that found in Colo 205 cells upon adhesion activation [28]. Further studies will be required to completely understand the mechanism regulating cadherin adhesive activity and how it relates to the function of adhesions in more complex systems.

## Materials and Methods

### Colo 205 Cell Culture and Activation Assay

Colo 205 cells (ATCC) were cultured in 5% CO_2_ at 37°C in DMEM/F12 with HEPES and L-glutamine (Gibco) supplemented with 10% FBS (Atlantic Biologicals). For activation assay, cells were collected by standard trypsinization and plated at 1.2 x 10^6^ cells/well in a 12-well plate and allowed to recover overnight. Spent media was carefully removed and replaced with fresh media during assay.

### Imaging

For live imaging of EB1-EGFP, cells were collected by standard trypsinization and 1.4 x 10^6^ cells were plated on a collagen-coated 35 mm glass-bottom dish (MatTek Corporation). After recovering overnight, the spent media was replaced with fresh media containing diluted DMSO/nocodazole. Each dish was treated and imaged separately at room temperature using an Eclipse TE2000 confocal microscope (Nikon). After imaging up to 15 min, treated plates were incubated in 5% CO_2_ at 37°C for an additional 1.75 hours. Adhesion activation was then analyzed via brightfield microscopy with images collected using a IX-71 microscope (Olympus), the same as similar assays in this paper. Cells were then fixed in -20°C methanol for 10 min, washed twice with PBS, then blocked for 30 min in 5% milk/PBST before overnight incubation with mouse anti-α-tubulin DM1A monoclonal antibody (Pierce, #62204) [1:2,500] at 4°C. Cells were then washed twice with PBS before incubation with rabbit anti-mouse IgG (H+L) 546 [1:500] in the dark for 2 hours at room temperature. Cells were washed twice and left in a third wash of PBS for imaging on an Eclipse TE2000 confocal microscope (Nikon).

### Chemicals

Nocodazole (Sigma, #M1404), paclitaxel or taxol (Sigma, #T7191), sodium chloride (Sigma, #S7653), lithium chloride (Sigma, #L4408), anisomycin (Cell Signaling Technologies, #2222), SB203580 (Cell Signaling Technologies, #5633), D4476 (Millipore/Calbiochem, #218696), Casein Kinase II Inhibitor I (Millipore/Calbiochem, #218697), PP2 (Sigma, #P0042), sorbitol (Sigma, #S1876), ciliobrevin D (Millipore/Calbiochem, #250401), skepinone-L (Millipore/Calbiochem, #506174), Serine/Threonine Kinase Inhibitor Set (Calbiochem, #539572) which includes bisindolylmaleimide I, H-89 dihydrochloride, KN-93, ML-7, and staurosporine.

### Retroviral Expression Constructs

Mouse wildtype p120 catenin isoform 3A and several mutants thereof (S252A, S268A, S288A, S312A, 6S/T>A) in the pLZRS-Neo retroviral vector were kind gifts from Albert Reynolds (Ireton *et al*., 2002; Xia *et al*., 2006). The 6S/T>A mutant includes S252A, S268A, S288A, T310A, S312A, and T910A mutations. The 4S/T>E mutant includes S268E, S288E, T310E, and S312E mutations. The 6S/T>E mutant includes S252E, S268E, S288E, T310E, S312E, and T910E. The 6S/T>D mutant includes S252D, S268D, S288D, T310D, S312D, and T910D. Additional mouse p120 mutants, including S268/S288A, T310A, T310/S312A, S268/T310/S312A, S252/T310/S312A, were created by overlap extension PCR using Phusion polymerase (NEB), digested with *EcoRI* and *SfiI*, and ligated to pLZRS-Neo. All viruses were produced using the Phoenix retrovirus producer cell line (Garry P. Nolan, Stanford University) according to the Nolan lab protocol. Colo 205 cells were infected with respective retroviruses by spinoculation in 6-well tissue culture plates at 1800 × *g* for 2 hr at 33°C and selected with 1 mg/ml neomycin for 7 days. Mouse p120 catenin expression levels were estimated by Western blot analysis using mouse p120–specific mAb 8D11 (Wu *et al*., 1998), a kind gift from Albert Reynolds. Human p120 catenin knockdown was achieved by electroporation of infected cells with human p120–specific siRNA (Davis *et al*., 2003) using Amaxa Nucleofector (Lonza) according to the manufacturer’s instructions.

For EB1-EGFP expression in Colo 205 cells, the cDNA from an existing plasmid (kind gift from George Bloom) was PCR amplified with Phusion polymerase, which added a 3' *SfiI* site, and digested with *EcoRI* and *SfiI* for ligation into pLZRS-Neo. Virus production and infection is same as above.

### Lentiviral Expression Constructs

Colo 205 cells with low E-cadherin expression (shRNA cells) were produced by E-cadherin shRNA-containing lentiviral infection. For infection, pLKO.1Puro plasmid from Addgene (Cat #18801, [61]) containing 5'-AAGATAGGAGTTCTCTGATGC-3' siRNA directed against E-cadherin was introduced into HEK293LT cells using Lipofectamine transfection. To produce a viable virus, pMD2.G envelop and psPAX2 packaging plasmids (Didier Trono, available on Addgene, Cat ## 12259 and 12260 respectively) were used according to Trono Lab protocol (http://tronolab.epfl.ch/). Colo 205 were infected with lentivirus by spinoculation at 3000 RPM for 2 hr at 33°C and selected with 10 μg/ml puromycin for 5 days. Selected cells were sub-cloned by limiting dilution and the clone with the lowest E-cadherin expression was expanded and used in all further experiments.

To address whether or not binding of p120 to E-cadherin is required to initiate adhesion activation, Colo 205 cells with low E-cadherin expression (shRNA cells) were further infected with lentivirus containing either wild type or EED>AAA E-cadherin mutant. EED>AAA mutation (E762A, E763A, D764A, pre-pro-protein sequence) was introduced into wild type E-cadherin by site-directed mutagenesis (QuikChange II XL Site-Directed Mutagenesis Kit, Agilent, Cat#200521). Then wild type and EED>AAA mutant E-cadherins were cloned into pLX304-Blast vector (Addgene, Cat # 25890) and used for lentivirus production together with pMD2.G envelop and psPAX2 packaging plasmids (see reference above). Colo 205 shRNA cells were infected by spinoculation as described above and selected with 10 μg/ml blasticidin for 10 days.

### SDS-PAGE and Western Blotting

For protein expression or phosphorylation analysis, cells were lysed in 1% triton X-100 with 50 mM Tris pH 7.4, 150 mM NaCl, 1 mM EDTA, cOmplete mini protease inhibitors EDTA-free (Roche Life Science), and PhosSTOP phosphatase inhibitor (Roche Life Science). Cells were incubated on ice for 10 min then vortexed 5 sec to break up cell clumps. Insolubles were pelleted at 10,000xg at 4°C in a tabletop microcentrifuge. The supernatant was collected and boiled for 5 min in SDS-DTT sample buffer. For protein expression analysis, samples were resolved by 6% SDS-PAGE. For phosphorylation analysis, samples were resolved by 6% SDS-PAGE using 37:5:1 bis-acrylamide with 20μM Phos-tag^®^ acrylamide and 10 mM MnCl_2_ running at 42 V. Gels were then washed twice for 10 min in transfer buffer supplemented with 10mM EDTA, and then twice for 10 min in transfer buffer alone. For both protein expression and phosphorylation analysis, proteins were transferred to PVDF membrane for 3 hours at 60 V. The membrane was blocked for 30 min in 5% milk/PBST and blotted overnight at 4°C with primary antibodies. After washing, blot was incubated with secondary antibodies for 45 min at room temperature. Blot was washed 3x in PBST and then imaged on a LI-COR Odyssey.

Primary antibodies used include rabbit anti-delta 1 catenin (p120-catenin) monoclonal (Abcam, ab92514) [1:2000], mouse anti-E-cadherin clone 36 monoclonal (BD Biosciences) [1:5,000], and mouse anti-α-tubulin DM1A monoclonal (Pierce, #62204) [1:2,500]. Secondary antibodies used include IRDye goat anti-mouse 800CW and goat anti-rabbit 680RD (LI-COR) [1:10,000].

### Subfractionation

Modified from Thoreson, *et*. *al*. [18]. Colo 205 cells were collected by standard trypsinization and plated at 1.2x10^6^ cells/well in a 6-well tissue culture dish. After recovering overnight, non-adherent cells in the media were gently pelleted and resuspended in 50 μL of ice-cold hypotonic buffer (10 mM Tris pH 7.4, 1 mM MgCl_2_, 1 mM EDTA, cOmplete mini protease inhibitors EDTA-free (Roche Life Science), and PhosSTOP phosphatase inhibitor (Roche Life Science)). Meanwhile, adherent cells were washed once with ice-cold PBS, then incubated in 200 μL ice-cold hypotonic buffer. The resuspended pellet was then added to the remaining cells in the dish and the rest of the protocol was done on ice. The cells were allowed to swell for 1 hr with frequent rocking. Cells were scraped and then broken by 20 strokes with a loose Dounce homogenizer followed by 80 strokes with a tight Dounce. 5 M NaCl was added to a final concentration of 0.15 M. Nuclei and unbroken cells were pelleted by a 3 sec spin at 14,000 rpm in a tabletop microcentrifuge. 150 μL of the supernatant was moved to a thick-walled ultracentrifugation tube (Beckman #343778) and pelleted at 100,000 x *g* for 30 min at 4°C in a TLA-120.2 rotor. The supernatant was collected carefully so as not to disturb the pellet and resuspended in 50 μL 4X SDS-DTT sample buffer (cytosolic fraction). The pellet was washed carefully in 150 μL ice-cold PBS and resuspended in 50 μL 1X SDS-DTT sample buffer (membrane fraction). All samples were boiled for 5 min then resolved by 7.5% SDS-PAGE, with a ratio of 1:4 membrane:cytosolic, and analyzed via Western blotting.

## Supporting Information

S1 Fig Adhesion activation in various phosphomimetic p120 mutants. Brightfield images correspond to summary in Table 2. The cells shown were treated with either 2 μg/mL 19A11 activating E-cadherin Fab, 10 μM nocodazole, or 55 mM LiCl. (A-D) Colo 205 cells with human p120 siRNA knockdown. (E-H) Colo 205 cells with human p120 siRNA knockdown plus expression of mouse p120-3A wildtype. (I-L) Colo 205 cells with human p120 siRNA knockdown plus expression of mouse p120-3A 6S/T>A mutant. (M-P) Colo 205 cells with human p120 siRNA knockdown plus expression of mouse p120-3A 6S/T>D mutant. (Q-T) Colo 205 cells with human p120 siRNA knockdown plus expression of mouse p120-3A 4S/T>E mutant.
(TIF)

S1 Table Colo 205 chemical treatment details. Data correspond to brightfield images in Fig 2.
(PDF)
