# Supplementary figures and images for: Microtubules Inhibit E-Cadherin Adhesive Activity by Maintaining Phosphorylated p120-Catenin in a Colon Carcinoma Cell Model
Source: PLoS One. 2016 Feb 4;11(2):e0148574. doi: 10.1371/journal.pone.0148574 (PMC4742228; doi:10.1371/journal.pone.0148574)

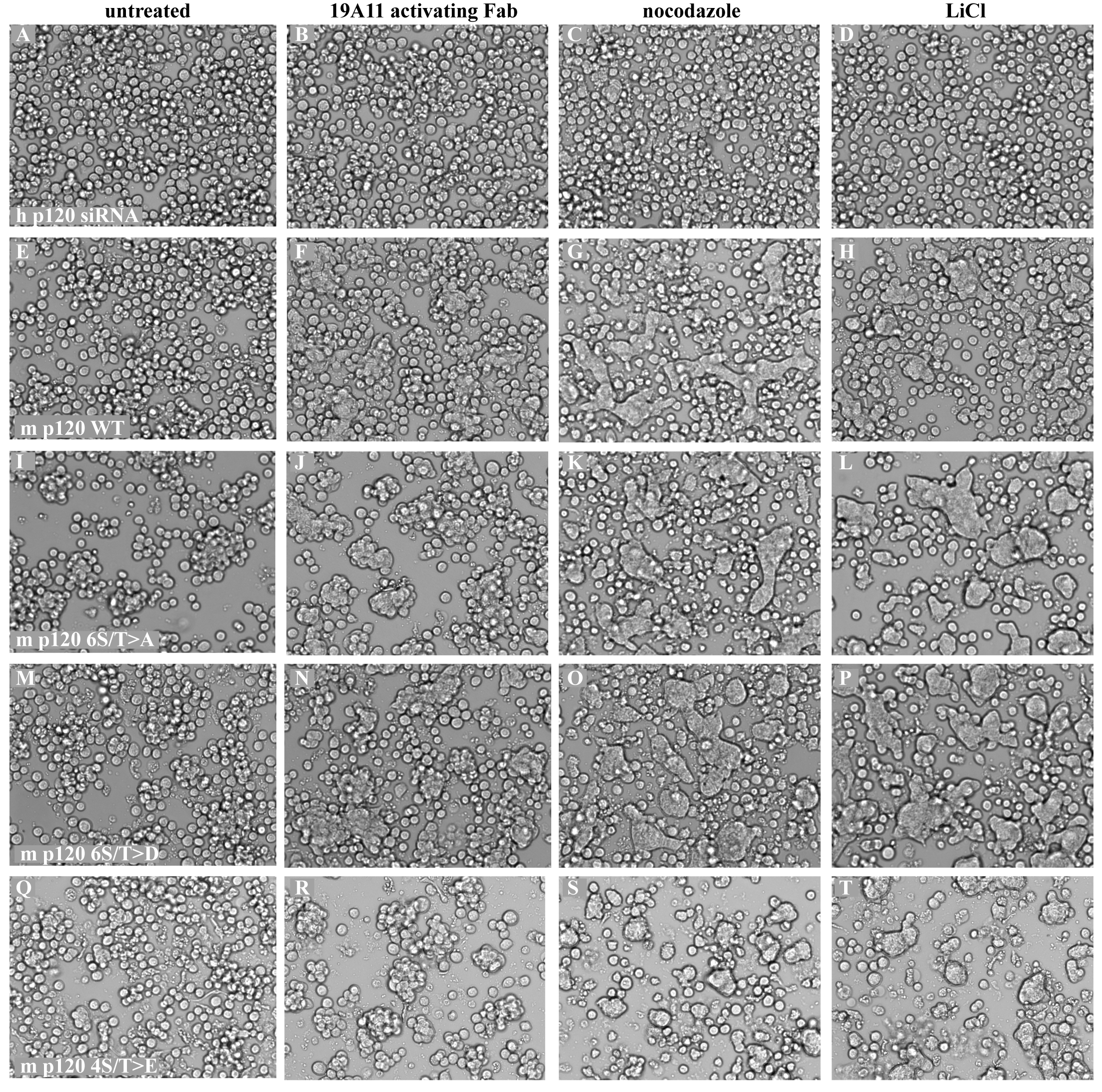

Supplement: S1 Fig — Brightfield images correspond to summary in Table 2. The cells shown were treated with either 2 μg/mL 19A11 activating E-cadherin Fab, 10 μM nocodazole, or 55 mM LiCl. (A-D) Colo 205 cells with human p120 siRNA knockdown. (E-H) Colo 205 cells with human p120 siRNA knockdown plus expression of mouse p120-3A wildtype. (I-L) Colo 205 cells with human p120 siRNA knockdown plus expression of mouse p120-3A 6S/T>A mutant. (M-P) Colo 205 cells with human p120 siRNA knockdown plus expression of mouse p120-3A 6S/T>D mutant. (Q-T) Colo 205 cells with human p120 siRNA knockdown plus expression of mouse p120-3A 4S/T>E mutant. (TIF) [file pone.0148574.s001.tif]
