# Supplementary material for: Microtubules Inhibit E-Cadherin Adhesive Activity by Maintaining Phosphorylated p120-Catenin in a Colon Carcinoma Cell Model
Source: PLoS One. 2016 Feb 4;11(2):e0148574. doi: 10.1371/journal.pone.0148574 (PMC4742228; doi:10.1371/journal.pone.0148574)
Supplement: S1 Table — Data correspond to brightfield images in Fig 2. (PDF) [file pone.0148574.s002.pdf]

S1 Table. Colo 205 chemical treatment details.

Target effect	Chemical	Concentration	Treatment length	Adhesion activated	p120 dephospho
-	-	-	-	**X**	**X**
none	DMSO	-	-	**X**	**X**
broad ser/thr kinase inhibition	staurosporine	0.1 µM	5 hrs	✔	✔
PKC inhibition	Bisindolylmaleimide I	100 µM	5 hrs	**X**	**X**
PKA inhibition	H-89 dihydrochloride	10 µM	5 hrs	**X**	**X**
CaMKII inhibition	KN-93	100 µM	5 hrs	**X**	**X**
MLCK inhibition	ML-7	10 µM	5 hrs	**X**	**X**
CK2 inhibition	casein kinase II inhibitor I	100 µM	2 hrs	**X**	**X**
Src family inhibition	PP2	10 µM	2 hrs	**X**	**X**
dynein transport inhibition	ciliobrevin D	100 µM	6 hrs	slight	**X**
salt control	NaCl	55 mM	1.5 hrs	**X**	**X**
GSK3 inhibitor	LiCl	55 mM	1.5 hrs	✔	✔
CK1 inhibition	D4476	50 µM	3 hrs	✔	**X**
p38 MAPK inhibition	SB203580	20 µM	3 hrs	✔	✔
p38 MAPK inhibition	skepinone-L	40 µM	2 hrs	**X**	**X**
p38 MAPK activation	anisomycin	12 µg/mL	5 hrs	✔	✔
p38 MAPK activation	sorbitol	200 µM	1.5 hr	✔	✔
p38 inhibition/ activation	skepinone-L + sorbitol	40 µM + 200 µM	2 hrs + 1.5 hrs	partial	ND

Treatment details related to Fig. 2 in the main text. For chemicals that did not activate adhesion, the highest dose and longest time point tested are shown. For those that did activate adhesion, the lowest effective dose and shortest effective time point are shown.
